# Analysis of LRP1 gene mutation in developmental dysplasia of the hip: a case series

**DOI:** 10.1186/s12920-026-02316-7

**Published:** 2026-01-24

**Authors:** Tianze Cheng, Weiling Zhang, Hui Cheng

**Affiliations:** 1 Haidian Foreign Language Academy, Beijing, 100195 China; 2 Senior Department of Orthopaedics, the Fourth Medical Center of PLA General Hospital, Beijing, 100048 China

**Keywords:** Developmental dysplasia of the hip (DDH), LRP1 gene, Genetic variability, Mutation analysis, DNA sequencing, Genotype‒phenotype correlation, Case report

## Abstract

Developmental dysplasia of the hip (DDH) is a multifactorial disorder that affects 0.56–3.8% of newborns worldwide. Recent research has identified several candidate genes potentially involved in DDH pathogenesis, with LRP1 investigated as a candidate gene due to its regulatory role in cartilage development. This study presents a genetic analysis of two female DDH patients (17 and 26 years old) diagnosed through radiographic examination. Genomic DNA was extracted from peripheral blood samples of the two female patients with DDH. We performed targeted genetic analysis of LRP1 exons 6, 32, 40, and 74, which have been previously implicated in DDH pathogenesis. DNA was extracted from peripheral blood samples, amplified via polymerase chain reaction (PCR), and analyzed using Sanger sequencing. Despite clear clinical DDH diagnoses, neither patient carried mutations in the examined LRP1 exons and displayed only wild-type sequences. These findings indicate that no pathogenic LRP1 variants were detected in these two DDH patients. This case report provides preliminary descriptive data on LRP1 exonic regions in DDH. The absence of detected variants in these specific loci suggests that future investigations should utilize high-throughput sequencing strategies in larger cohorts to more comprehensively explore the genetic basis of the disorder.

**Supplementary Information:**

The online version contains supplementary material available at 10.1186/s12920-026-02316-7.

## Introduction

Developmental dysplasia of the hip (DDH) is a developmental disorder characterized by various structural abnormalities of the hip joint, resulting in morphological alterations of the femoral head and acetabulum, as well as ligamentous laxity [1–4]. The prevalence of DDH ranges from 1 to 2/1000 in unscreened populations to 5–30/1000 in clinically screened populations, affecting approximately 0.56%–3.4% of newborns worldwide [5, 6]. Management of DDH varies depending on the disease stage and may include both nonsurgical and surgical interventions, with early diagnosis being crucial for optimizing treatment outcomes [7].

With advances in genetic technology, our understanding of DDH pathogenesis has evolved from purely mechanical perspectives to recognition of its complex genetic architecture. Several candidate genes potentially associated with DDH have been identified, including the HOX gene family (HOXA, HOXB, and HOXD), which specifically regulates biological morphology [8, 9] and can cause pathological changes in body segments when mutated; collagen-related genes (COL1A1 and COL1A2) that control cartilage development [10, 11] growth differentiation factor 5 (GDF5), the first gene discovered to be associated with DDH pathogenesis, whose recombinant protein promotes bone regeneration and cartilage repair and is used to treat degenerative joint changes [12–15] estrogen receptor (ER) [16] and DLX family genes (DLX3, DLX4, and DLX5) [9, 17, 18], among others [19, 20].

Among these candidates, we selected the LRP1 gene for investigation. LRP1 plays a critical role in normal hip joint development through multiple mechanisms, including proliferation, differentiation, metabolism, regulation of the timing of hip cartilage development, maintenance of chondrocyte differentiation capacity, and balancing autophagy with β-catenin signaling pathways [21, 22]. Genetic variants or functional defects in LRP1 can disrupt these mechanisms, ultimately leading to DDH. As a recently discovered gene associated with DDH, LRP1 has significant research value. Key point mutations such as c.5347 C > T [p.R1783W] and c.6386 C > A [p.T2129K] have been shown to eliminate LRP1 function [22].

We aimed to examine specific exons of the LRP1 gene in DDH patients to assess the presence of variants in these functionally important regions.

## Case report

**Fig. 1:**
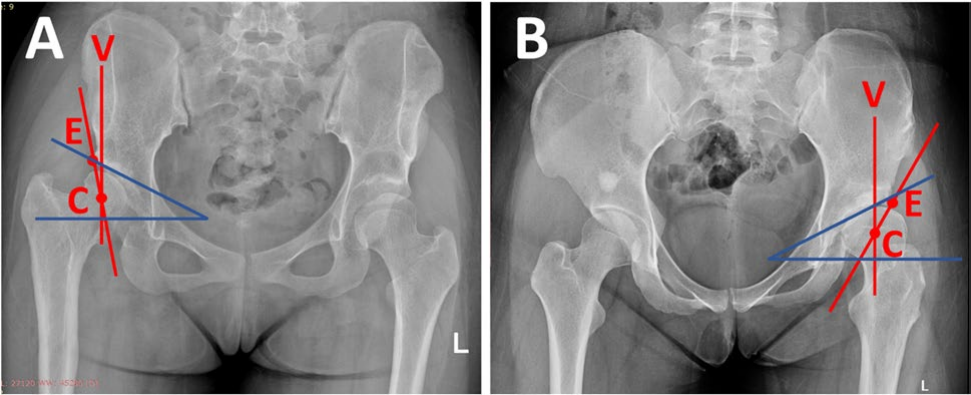
Anteroposterior pelvic radiographs of both study participants. Point C indicates the center of the femoral head, whereas point E denotes the lateral acetabular edge. The line CV represents a vertical reference through the center of the femoral head. The angle between lines CE and CV constitutes the lateral center-edge angle (LCEA). The oblique blue line connects the acetabular margins (medial to lateral), and the horizontal blue line serves as the reference plane. The angle formed between these blue lines represents the Tönnis angle (TA). **A** Patient 1, (**B**) Patient 2

 We report two female patients who were diagnosed with developmental dysplasia of the hip (DDH) and who were scheduled for periacetabular osteotomy (PAO) at our institution. Both cases were diagnosed through radiographic evaluation using lateral center edge angle (LCEA) and Tonnis angle (TA) measurements (Fig. 1A and 1B).

Patient 1 was a 17-year-old female who presented with a one-year history of right hip pain. Her anthropometric measurements were weight 50 kg, height 159 cm, and BMI 23.3 kg/m². Radiographic assessment revealed an LCEA of 11.6° and a TA of 23.2°, confirming the diagnosis of DDH.

Patient 2 was a 26-year-old female with a 20-year history of limping that had worsened over the past year. Her anthropometric measurements were weight 60 kg, height 162 cm, and BMI 22.9 kg/m². Radiographic assessment revealed an LCEA of 0° and a TA of 25°, which is consistent with severe DDH.

Neither patient reported a family history of infectious or hereditary disorders or previous surgery on the affected hip, and both sets of parents were healthy. Following clinical diagnosis, we performed targeted genetic analysis of LRP1 exons 40, 32, 74, and 6 [22]. These specific exons were selected on the basis of previous research identifying functionally critical variants in these regions, including c.5347 C > T (p.R1783W) in exon 32 and c.6386 C > A (p.T2129K) in exon 40, which have been shown to cause loss of function in LRP1. These regions encode highly conserved domains essential for LRP1’s role in regulating autophagy and β-catenin signaling during chondrocyte differentiation and triradiate cartilage development. Sequencing was conducted using a combination of Sanger sequencing and next-generation sequencing technologies. The results for both patients were negative and revealed wild-type sequences in all the examined exons with no mutations detected. The sequencing chromatograms showing wild-type sequences are provided in the supplementary materials. These findings indicate that despite clear clinical DDH diagnoses, neither patient carried mutations in the target exons of the LRP1 gene previously associated with the pathogenesis of this condition.

## Methods

This study was designed as an exploratory case series limited to clinically confirmed DDH patients. A healthy control group was not included because this preliminary investigation focused on descriptive sequence verification rather than statistical association analysis or risk comparison.

### Primer design

In this study, primer design for specific exonic regions of the LRP1 gene was based on literature retrieved from the National Library of Medicine (NCBI) database. To ensure primer specificity and optimal amplification efficiency, all the designed primer sequences were evaluated and validated using the NCBI Primer-BLAST tool. Specific primers targeting exons 6 (670), 32 (5347), 40 (6386), and 74 (11441) of the LRP1 gene were synthesized by Sangon Biotech (Shanghai) Co., Ltd. All primer designs considered the optimal G and C contents, annealing temperature, self-complementarity, and potential secondary structure formation to ensure high efficiency and specificity of polymerase chain reaction (PCR) (see Table 1).

**Table 1:** Target gene primer sequences for PCR amplification

Target gene	Primer Sequence
670 F	CGGCTCCTTCATATGTGGCT
670R	TAACTCCCGGCCTCTGTTCA
5347 F	GTCCTTCAGTTGCCCCTCAG
5347R	CCCATTGGCCCTAAGTCATCA
6386 F	ATCAGCCTCACAGGTCCATTC
6386R	TGTGACGAGGCATTGCACTT
11,441 F	ATACCCAGGGCCTAAAGCCT
11441R	GGCCCCGTTTGATATCCTGG

The numbers in the primer names represent the target nucleotide positions

*F* Forward primer, *R* Reverse primer, *PCR* Polymerase chain reaction, *A* Adenine, *T T*hymine, *C* Cytosine, *G* Guanine

### Genomic DNA isolation

Genomic DNA was extracted from peripheral blood samples of the two female patients with DDH. A commercial DNA extraction kit (ZOMANBIO, Beijing, China) was used, with slight modifications to the standard extraction protocol according to the experimental requirements. The specific procedure was as follows: 250 μl of fresh frozen blood sample was transferred to an Eppendorf tube, mixed thoroughly with 10 μl of proteinase K solution, and incubated in a 70 °C water bath for 10 minutes. Afterward, 250 μl of Buffer B was added and gently mixed, followed by continued incubation at 70 °C for 10 minutes. After 250 μl of anhydrous ethanol was added, the mixture was vortexed for 15 seconds to form a flocculent precipitate. The mixture was transferred to an adsorption column, and after adding 500 μl of Buffer C, it was centrifuged at 12,000 rpm for 30 seconds and the flow-through was discarded. This was followed by two washing steps with 700 μl of Wash Buffer W2, each time centrifuging at 12,000 rpm for 30 seconds and discarding the flow-through. Finally, the adsorption column was centrifuged for an additional 2 minutes to remove residual wash solution, air-dried at room temperature, and transferred to a new centrifuge tube, after which 100 μl of TE elution buffer was added and incubated at room temperature for 2–5 minutes, followed by centrifugation at 12,000 rpm for 2 minutes to collect the DNA solution. The concentration and purity of all the extracted DNA samples were measured using a spectrophotometer, with Sample 2 showing an optimal DNA concentration, while Sample 1 had a concentration slightly below the ideal range but was still suitable for subsequent PCR amplification.

### PCR amplification

PCR amplification was performed on the exonic regions of the LRP1 gene for two DNA samples. Prior to PCR, the lyophilized primers were centrifuged at 4000 rpm for 60 s and then reconstituted with nuclease-free water to a final concentration of 100 µM. Each amplification reaction had a total volume of 50 µl, containing 25 µl of PCR master mix (including buffer, 6× loading dye, Mg²⁺, and DNA polymerase), template DNA (2 µl for sample 1 and 1 µl for sample 2 because of their concentration differences), 1 µl of primer mixture (containing 0.5 µl of forward primer and 0.5 µl of reverse primer), and an appropriate amount of nuclease-free water (22 µl for sample 1 and 23 µl for sample 2). Separate reactions were performed for exons 6, 32, 40, and 74 of the LRP1 gene, with a total of 12 reaction tubes prepared for the two DNA samples. After brief centrifugation, the reaction mixtures were placed in a thermal cycler for 35 cycles of amplification. The PCR amplification program included initial denaturation (95 °C for 2 min); 35 cycles of denaturation (94 °C for 30 s), annealing (53 °C for 30 s), and extension (72 °C for 1 min); and a final extension, with specific temperature and time parameters optimized for different primer pairs. To validate the accuracy of our PCR system, we performed PCR amplification of the ACTIN gene using DNA templates from both samples. The expected product size was 866 bp, which was compared against a no-template control (NTC) to confirm the specificity of our PCR amplification.

### Assessment of PCR product specificity

**Fig. 2:**
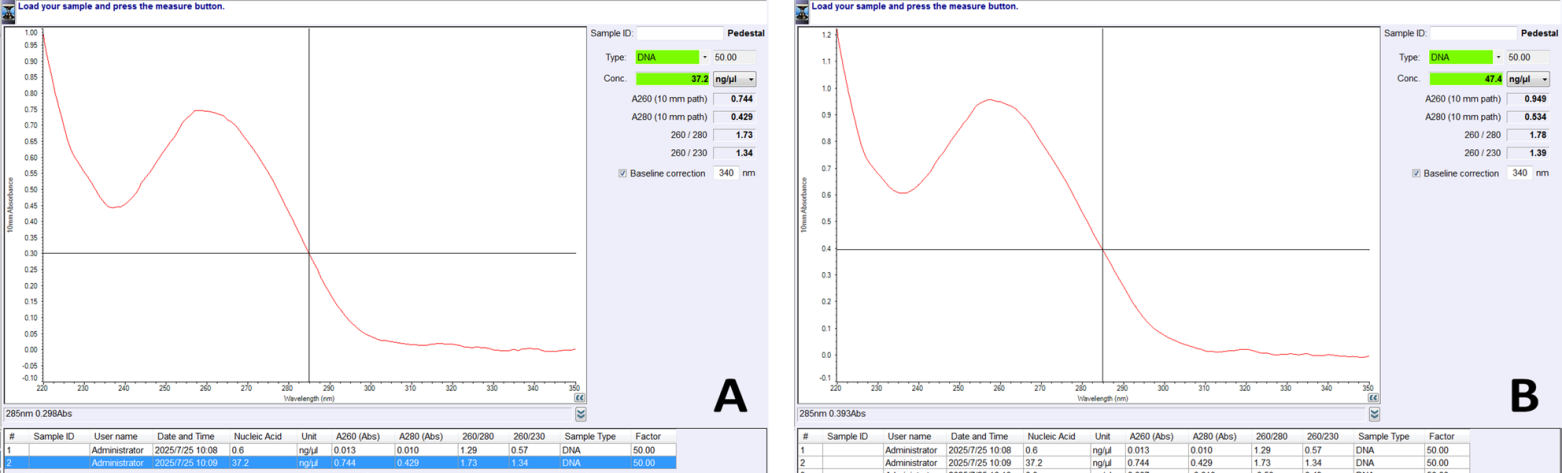
DNA quantification analysis performed using a Nano800 Ultra-Micro Nucleic Acid and Protein Detector. (**A**) Quantification results for Patient 1, (**B**) Quantification results for Patient 2.

To assess the specificity of the PCR amplification products, this study employed agarose gel electrophoresis for analysis. An agarose gel with 25 loading wells was prepared, with one well loaded with a DNA molecular weight marker to determine the base pair size of the PCR products, and the remaining 24 wells were loaded with different PCR amplification products. After electrophoresis, the gel was visualized using a nucleic acid gel imaging system. Preliminary electrophoresis revealed nonspecific amplification in some PCR products, characterized by multiple DNA bands appearing in a single loading well (as shown in Fig. 2.a), indicating that the PCR conditions required further optimization. Ideal PCR amplification should appear as a single clear band in gel imaging, demonstrating that the primers specifically bound to and amplified the target sequence. Nonspecific amplification may be caused by factors such as primer design, unsuitable annealing temperature, or excessive template DNA concentration.

### PCR condition optimization

**Fig. 3:**
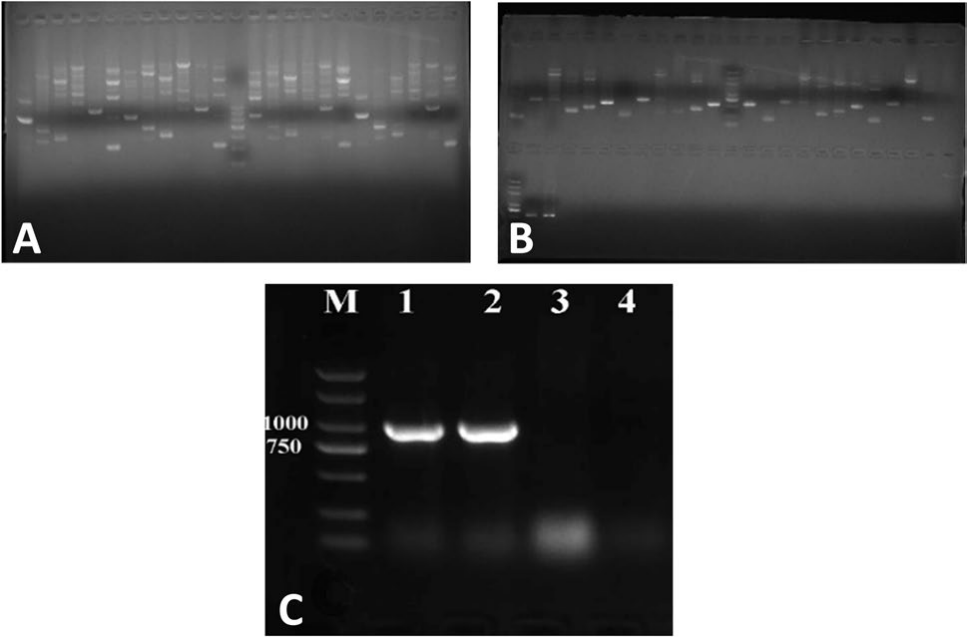
Comparative sequence alignment demonstrating the absence of variations between the LRP1 gene sequences obtained from both patients (represented in the left panel and yellow regions in the right panel) and the reference human genome sequence (displayed in the orange region of the right panel)Figure 3. Electrophoretic analysis of PCR amplification across four exons at different annealing temperatures. (**A**) PCR products from Patient 1 and Patient 2 amplified at 50 °C and 53 °C; DNA size marker is included. (**B**) PCR products from Patient 1 and Patient 2 amplified at 55 °C and 58 °C, including reactions using diluted DNA template and corresponding DNA size markers. (**C**) Amplification of the ACTB gene (866 bp) as a positive control. M, DNA marker; lanes 1–2, patient samples; lanes 3–4, no‑template controls (NTC).

On the basis of the nonspecific amplification observed in the preliminary PCR results, this study systematically optimized the PCR conditions. The main optimization strategies included increasing the annealing temperature and adjusting the amount of template DNA. Specifically, the annealing temperature was increased from 50 to 53 °C to 55–58 °C, while the other PCR components remained unchanged. For sample 2 with a higher DNA concentration, additional testing was performed using 0.5 µl of template DNA (with a corresponding increase of 0.5 µl of nuclease-free water) at both 55 °C and 58 °C annealing temperatures. Under these optimized conditions, exons 6, 32, 40, and 74 of the LRP1 gene were reamplified, with a total of 14 reaction mixtures prepared. After amplification, two agarose gels (each containing 25 loading wells) were used for electrophoresis analysis, with the gels prepared using 0.6 g of agarose powder, 70 ml of buffer, and 7 µl of nucleic acid dye. After the gel cooled, 10 µl of amplification product was loaded into each well and electrophoresed at an appropriate voltage for 20 min. The optimized gel imaging results (as shown in Fig. 3b) demonstrated that most of the target exonic regions yielded amplification products with sufficient specificity for downstream DNA sequencing analysis.

### DNA sequencing and DNA sequence analysis

PCR products with successful specific amplification were sent to Sangon Biotech (Shanghai) Co., Ltd., for Sanger sequencing. The sequencing reaction employed a bidirectional sequencing strategy to ensure the accuracy and reliability of the sequence data. Quality control was performed by comparing forward and reverse sequencing results for each sample, ensuring concordance between both strands before proceeding with analysis. The obtained sequencing data were analyzed using professional bioinformatics software, including sequence quality assessment, base identification, and alignment analysis. Sequencing was performed bidirectionally (both forward and reverse strands), with concordant results. The LRP1 gene sequencing results from DDH patient samples were compared with the human reference genome sequence in the NCBI database to identify potential sequence variations. The alignment analysis focused on nucleotide substitutions, deletions, insertions, or other sequence variations potentially related to the pathogenesis of DDH. The sequence alignment and variation analysis results were used to evaluate the potential association between LRP1 gene mutations and DDH.

### Sequence alignment with the reference genome

Forward and reverse sequencing chromatograms were aligned with the human reference genome (NCBI database, RefSeq accession number for LRP1). Patient sequences (displayed on the left panels) were compared position-by-position against reference sequences (displayed on the right panels) at nucleotide positions 670, 5347, 6386, and 11,441. Sequence variations, including SNPs, insertions, and deletions, were documented.

### Ethics statement

This study was conducted in accordance with the Declaration of Helsinki and approved by the Institutional Review Board of the Fourth Medical Center of Chinese PLA General Hospital (Approval Number: 2023KY002-KS001). Written informed consent was obtained from all participants or their legal guardians prior to enrollment. Patient confidentiality was maintained throughout the research process, with all personal identifiers removed during analysis and reporting. All biological samples were collected using standard clinical procedures with minimal discomfort to patients, who were informed of their right to withdraw from the study at any time without affecting their clinical care.

## Results

### Demographic characteristics of the study participants

This case series included two patients diagnosed with developmental dysplasia of the hip (DDH). Both Patient 1 and Patient 2 were diagnosed with DDH through comprehensive clinical evaluation and radiological examination, but their condition was not influenced by the key loci of these nucleotide positions.

### Optimization of PCR amplification conditions

Initial PCR amplification at annealing temperatures of 50 °C and 53 °C produced multiple nonspecific bands, indicating suboptimal PCR conditions. Subsequently, increasing the annealing temperature to 55 °C and 58 °C significantly reduced nonspecific amplification. Electrophoretic visualization of PCR amplification products across four exons at varying annealing temperatures was then conducted as follows: (A) Lanes 1–6: Patient 1 at 50 °C; Lanes 7–12: Patient 2 at 50 °C; Lane 13: DNA size standard; Lanes 14–19: Patient 1 at 53 °C; Lanes 20–25: Patient 2 at 53 °C. (B) Lanes 1–6: Patient 1 at 55 °C; Lanes 7–12: Patient 2 at 55 °C; Lane 13: Diluted DNA template; Lanes 14 and second row Lane 1: DNA size standards; Lanes 15–20: Patient 1 at 58 °C; Lanes 21–25 and second row Lane 2: Patient 2 at 58 °C; Second row Lane 3: PCR product derived from the diluted template, as shown in Fig. 2A and B.

To validate the accuracy of our PCR system, we analyzed the ACTIN gene amplification results by gel electrophoresis, as shown in Fig. 2C. The ACTIN gene (organism: *Homo sapiens*; gene: ACTB) has a DNA sequence length of 3454 bp. The primers used were as follows: forward primer (ACTBF), CGCCCTTTCTCACTGGTTCT; reverse primer (ACTR), GGGTAACCCTCATGTCAGGC, generating a product size of 866 bp with a melting temperature (Tm) of 60 °C. These results confirm the validity of our PCR amplification system. Wells 1 and 2 contained DNA templates from Case 1 and Case 2, respectively, while wells 3 and 4 served as no-template controls (NTCs). Clear bands were observed in wells 1 and 2, whereas no significant bands appeared in wells 3 and 4. The numbers on the side indicate the molecular weight marker. Wells 1 and 2, containing the DNA templates from our two samples, successfully amplified the ACTIN gene product at 866 bp, serving as a positive control and confirming the validity of our amplification system. Wells 3 and 4, which contained no DNA template, served as negative controls, demonstrating that the amplification in wells 1 and 2 was specific. These results collectively confirm the reliability of our PCR system.

This optimization successfully generated specific PCR products suitable for downstream sequencing analysis. The improved protocol allowed for precise amplification of the target LRP1 gene regions, ensuring reliable sequencing data.

**Fig. 4:**
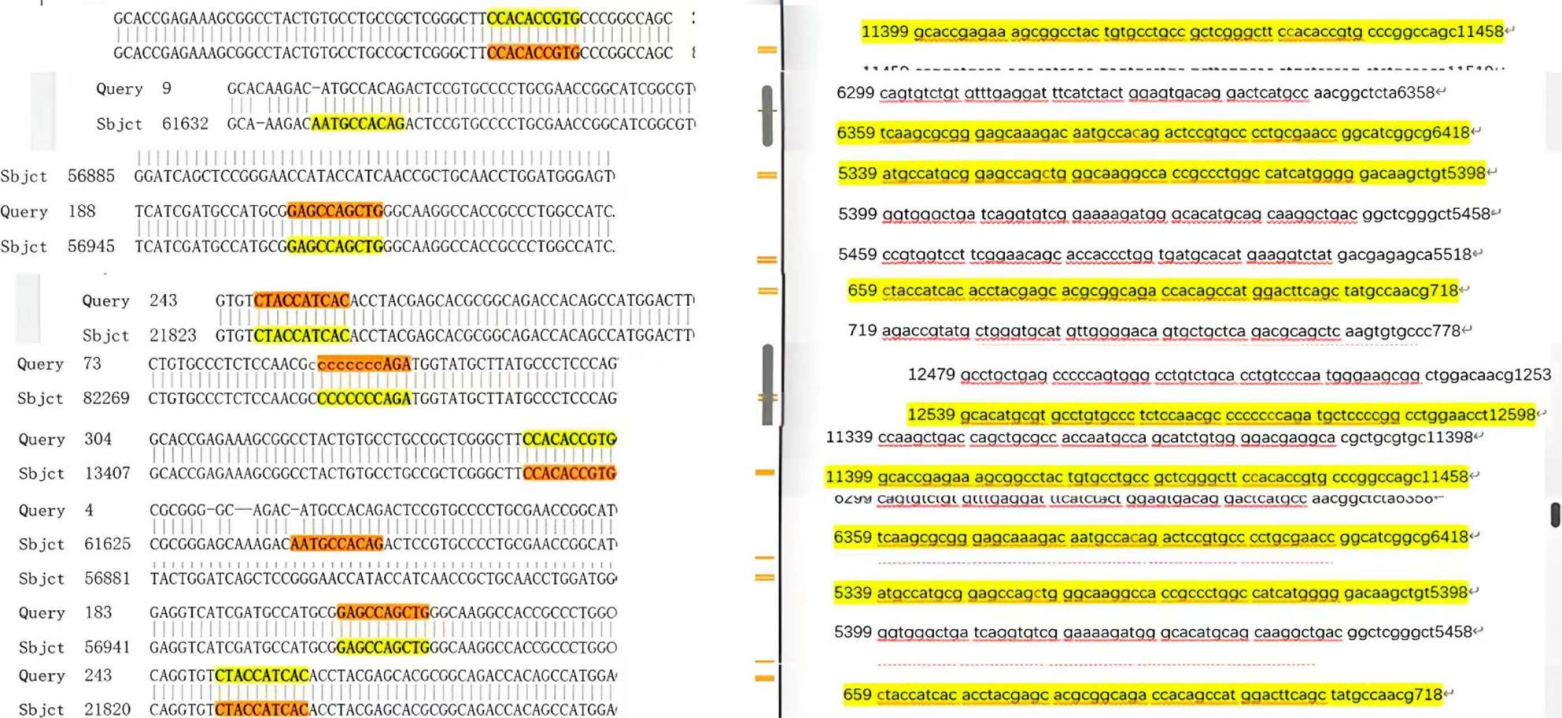
Comparative sequence alignment of the LRP1 gene sequences obtained from both patients and the reference human genome sequence. Highlighted regions indicate identical nucleotide sequences, demonstrating the absence of detectable variations.

When the DNA sequences of the two DDH patients were compared with the reference genome in the NCBI database, no mutations were found in the examined regions of the LRP1 gene (Fig. 4). No single-nucleotide polymorphisms (SNPs), insertions, or deletions were detected in any of the examined LRP1 regions in either patient. Specifically, four key regions of the LRP1 gene (region 11441, region 6386, region 5347, and region 670) were thoroughly sequenced in patient 1 and patient 2, and the sequences showed concordance with the reference genome in these regions. These findings remained consistent across all the examined LRP1 regions in both patients.

### Comparison with the reference genome

By comparing the nucleotide bases at specific loci in the gene sequences of the two investigated patients (left side of the figures) with those in the normal human reference genome sequence (right side of the figures), no differences were detected.

These sequencing results demonstrate complete concordance between the patient samples and the reference genome at all the loci examined, indicating the absence of mutations in these specific regions of the LRP1 gene in both DDH patients.

## Discussion

Through sequencing analysis of the LRP1 gene in 2 DDH patients, this study revealed no significant differences between the target gene sequences and the reference genome. These results suggest that mutations in the four examined regions of the LRP1 gene were not present in these two DDH cases, indicating that variations in these specific regions may not be universal contributors to DDH pathogenesis. The findings of this study provide a new perspective for exploring the complex genetic mechanisms of DDH, supporting the view that multiple gene interactions and environmental factors jointly contribute to DDH development. Additionally, regarding PCR optimization for the LRP1 gene, the primer melting temperature (Tm) was calculated to be 58 °C. Following the standard PCR protocol, the initial annealing temperature was set at 53 °C (Tm minus 5 °C). However, our optimization experiments demonstrated that 58 °C yielded the most specific amplification results, as shown in Fig. 2.b.

This study has several limitations. First, the small sample size (only 2 patients) limits the general applicability of the research results. Second, we sequenced only four key regions of the LRP1 gene, potentially missing other regions where variations might exist. Additionally, this study did not include a healthy control group for comparative analysis, nor did it examine changes in LRP1 gene expression levels. Finally, by focusing only on a single gene, we were unable to comprehensively evaluate other candidate genes potentially involved in DDH pathogenesis and their interactions with LRP1. Any potential implications for genetic counseling or predisposition assessment would require larger, hypothesis‑driven cohort studies with family‑based designs.

From a molecular mechanism perspective, our sequencing results revealed no sequence variations in the four regions of the LRP1 gene in either DDH patient. As a multifunctional endocytic receptor, LRP1 participates in the regulation of various biological processes, including lipid metabolism, cell signaling, and extracellular matrix remodeling [23–25]. Background studies on other complex diseases have highlighted shared genetic frameworks and pleiotropic patterns, providing a broader methodological perspective [26–28]. In cartilage development, LRP1 influences the differentiation and maturation of chondrocyte precursor cells by modulating the signaling of growth factors (such as TGF-β and BMP) [29].

Our PCR optimization process ensured highly specific amplification, and the sequencing chromatograms displayed clear peak patterns, confirming the reliability of our results. Through systematic comparison with the reference genome (Fig. 3), we demonstrated complete sequence concordance at nucleotide positions 670, 5347, 6386, and 11,441 in both patients. The forward and reverse sequencing chromatograms revealed identical base sequences to the reference genome at all the loci examined, with no single nucleotide polymorphisms (SNPs), insertions, or deletions detected. These findings demonstrate that no pathogenic variants were detected in the LRP1 exonic regions examined in the two DDH patients analyzed. As an illustrative analogy from hematology, incomplete penetrance and polygenic susceptibility have been commonly described in hereditary disorders, where disease manifestation depends on the cumulative effects of multiple genetic variants and environmental factors rather than single gene mutations [30–32]. On the basis of our sequencing analysis results, we propose that DDH likely follows a multifactorial etiological model. Although no variations were detected in LRP1 in either patient, the occurrence of DDH may involve the synergistic influence of multiple gene interactions and environmental factors [33, 34]. Epidemiological evidence suggests that factors such as intrauterine constraints (e.g., breech presentation and oligohydramnios) and postnatal positioning practices (e.g., tight swaddling) can increase the risk of DDH [35–37], which is consistent with the role of mechanical influences on acetabular and femoral head development. These environmental effects may also modulate penetrance in genetically susceptible individuals [38]. For instance, variations in other candidate genes, such as HOX family genes and collagen-related genes, or environmental factors, such as intrauterine constraint and improper swaddling, may interact with the LRP1 gene to affect hip joint development. This model explains why DDH phenotypes observed clinically exhibit high heterogeneity and why familial clustering does not always follow simple Mendelian inheritance patterns. This view is supported by our experimental results, which show that individuals carrying normal LRP1 gene sequences can still develop DDH.

From a clinical application perspective, our study results have important implications for genetic counseling and risk assessment of DDH. Our findings suggest that screening only the examined regions of LRP1 would not identify risk factors in all DDH patients, supporting the need for comprehensive multigene analysis. Our data indicate that patients can present with typical clinical manifestations of DDH even with normal sequencing results in all four key regions. Furthermore, this finding provides new insights for treatment strategies, suggesting that interventions targeting downstream signaling pathways (such as the Wnt/β-catenin pathway) may be more effective than simply targeting LRP1. Future research should explore the synergistic effects of LRP1 with other cartilage-related genes (such as GDF5 and the DLX family), as well as functional interactions at the protein level, to construct a more complete network of DDH pathogenesis mechanisms. Mendelian randomization has also been increasingly applied in genetic epidemiology as a hypothesis-driven approach to investigate potential causal relationships [26].

In conclusion, our study, through PCR optimization and targeted sequencing analysis of four key regions of the LRP1 gene in two DDH patients, revealed no pathogenic variants were identified in the examined LRP1 regions in these two DDH cases. (Fig. 3). Our present study should be interpreted as providing preliminary descriptive data. Owing to the small sample size and analysis of only partial regions of LRP1, future research needs to expand the sample size, perform whole-gene sequencing, and include healthy control groups to more comprehensively understand the role of LRP1 in the pathogenesis of DDH.

## Supplementary Information

Supplementary Material 1.

Supplementary Material 2

## Data Availability

The datasets generated during the current study are available from the National Genomics Data Center (NGDC) under accession number HRA014817.
