# Supplementary figures and images for: Analysis of LRP1 gene mutation in developmental dysplasia of the hip: a case series
Source: BMC Med Genomics. 2026 Jan 24;19:38. doi: 10.1186/s12920-026-02316-7 (PMC12911242; doi:10.1186/s12920-026-02316-7)

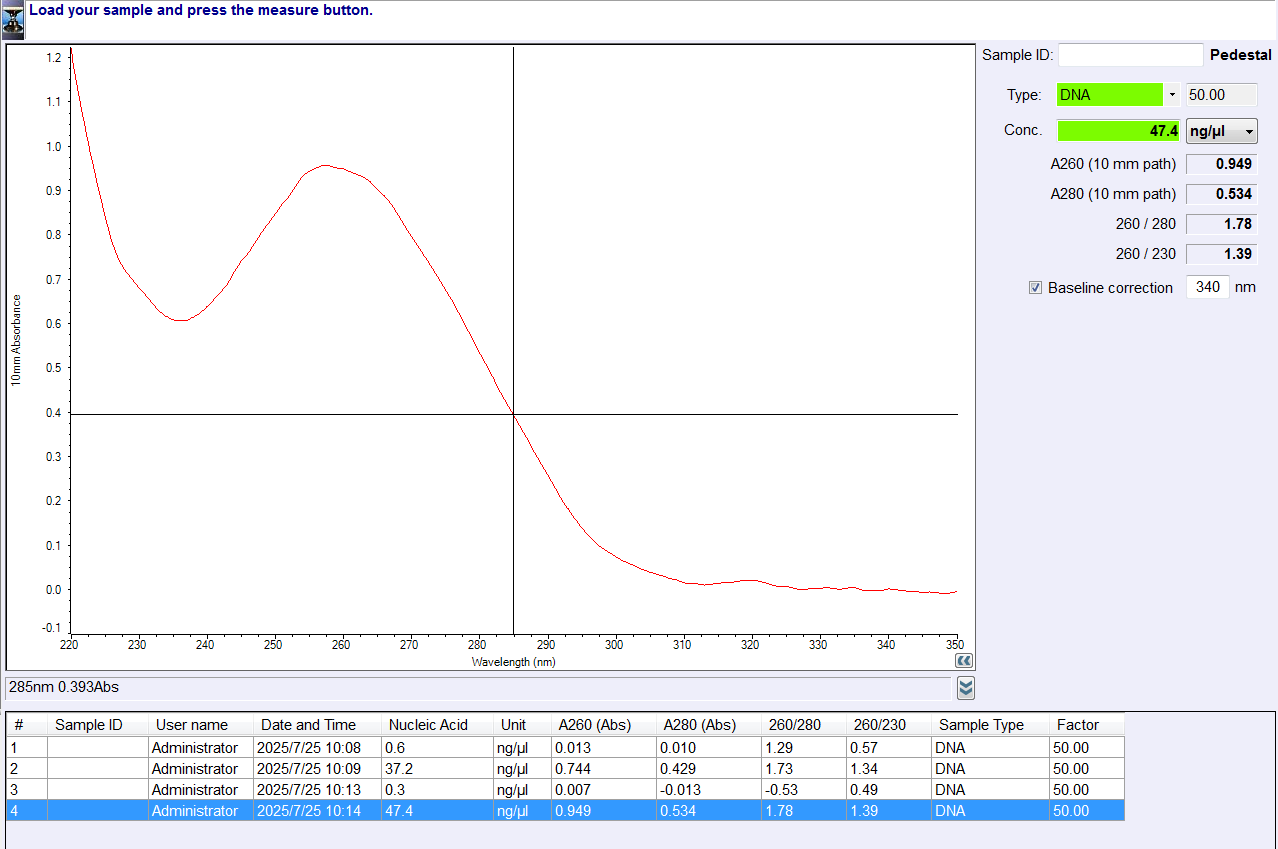

Supplement: Supplementary file 2 — Supplementary Material 2 [file 12920_2026_2316_MOESM2_ESM.png]
